# Patterns of lymph node metastasis in locally advanced cervical cancer

**DOI:** 10.1097/MD.0000000000004814

**Published:** 2016-09-30

**Authors:** Zhikai Liu, Ke Hu, An Liu, Jie Shen, Xiaorong Hou, Xin Lian, Shuai Sun, Junfang Yan, Fuquan Zhang

**Affiliations:** a Radiation Oncology, Peking Union Medical College Hospital; b Radiation Oncology, City of Hope Medical Center.

**Keywords:** cervical cancer, clinical target volume, lymph node metastasis

## Abstract

The aim of this study was to investigate patterns and locations of lymph node metastasis in locally advanced cervical cancers.

A total of 244 consecutive patients with stage IIb cervical cancer were retrospectively evaluated. Contrast-enhanced CT scans were used for lymph node grading. Lymph nodes with the shortest axis (>1 cm) were categorized as positive and those between 0.5 and 1 cm were categorized as suspicious. All lymph nodes (LNs) were also classified by their anatomic locations.

Nine hundred thirty-one LNs (136 positive and 795 suspicious) were identified. Sixty-three (25.8%) patients had positive LNs, and 153 (62.7%) patients had only suspicious LNs. The metastatic pattern was predictable traveling from level 1 (external iliac, internal iliac, obturator, and mesorectum groups) through level 2 (common iliac and presacral groups) to level 3 (para-aortic groups). In most groups, LNs were located within 1.0 cm of main blood vessels. Our novel findings were: presacral LNs metastases were rare (2/244, 0.82%); the left common iliac group (LCI) had significantly more enlarged nodes than the right common iliac group (*P* = 0.00); the LCI and left down-para-aortic group were further away from blood vessels than expected (1.2 cm and 1.4 cm, respectively); no additional margin was needed in anterolateral direction for external iliac groups.

The lymph node metastatic patterns are relatively predicable. Different expansions from vessels should be used to include LNs for different groups. Presacral nodes metastases are rare, and further study is warranted to see whether this region can be excluded from nodal CTV.

## 1 Introduction

Cervical cancer is one of the most common gynecologic malignant tumors. In developing countries, a significant portion of patients were locally advanced when diagnosed. Concurrent chemoradiotherapy is the standard treatment for locally advanced cervical cancer, and pelvic radiotherapy plays a key role in multimodality patient management. Intensity-modulated radiotherapy (IMRT) can reduce the high dose volume of small bowel, rectum, and bladder by 20% to 50% compared with conventional radiotherapy, and thus can significantly reduce toxicities.^[1–3]^ The widespread use of IMRT requires a clear understanding of the 3-dimensional locations of pertinent lymph node as well as its status and metastasis pattern. Accurate delineation of the nodal clinical target volume (CTV) is essential to deliver radiation to tumor cells while sparing critical organs nearby. In the era of conventional radiotherapy, the radiation fields were designed based on bony land markers. People believe lymph nodes (LNs) lay adjacent to major blood vessels, and CTV is typically defined by adding a margin around major blood vessels. Although several articles have been published to provide guidelines on how to contour CTVs,^[4,5]^ there is not enough detail on lymph node metastatic pattern and how to define CTV. For example, it is debatable that whether the use of a uniform margin around the blood vessels is the most appropriate method to define the CTV for nodal regions. Sample sizes in the published studies were small and very few studies were focused on locally advanced cervical cancer patients.

The purpose of this study was to evaluate the pattern on the lymph node distribution in a large group of locally advanced cervical cancer patients.

## 2 Materials and methods

### 2.1 Patient enrollment

From October 2012 to October 2014, 244 consecutive patients with locally advanced cervical cancer entered this study. According to the staging criteria of International Federation of Gynecology and Obstetrics (FIGO), all patients were stage IIB. We focused our study on IIB patients because they were the most common locally advanced cervical cancer patients in our center. IA, IB, and IIA patients are considered as early stage and surgery is often selected. IIIA and IIIB patients have lower vagina or pelvic wall involvement. The LNs metastatic patterns can be different from IIB patients. Mixture of IIB, IIIA, and IIIB patients may complicate the data uniformity. Therefore, we only included IIB patients in this study. However, the IIIA and IIIB patients will be analyzed and reported in another study. Median age of 244 patients was 50.5 years (range 26–81 years). All tumors were histologically diagnosed according to World Health Organization (WHO) classification: 215 cases of squamous cell carcinoma, 2 cases of adenosquamous carcinoma, and 27 cases of adenocarcinoma. All patients in this study received concurrent chemoradiotherapy (weekly cisplatin, 40 mg/m^2^, 4–6 cycles).

This study only involves the collection or study of existing data, documents, records, pathological specimens, or diagnostic specimens, and the information is recorded by the investigator in such a manner that subjects cannot be identified, directly or through identifiers linked to the subjects. The institutional review board of Peking Union Medical College Hospital (PUMCH) reviewed the protocol and approved the study.

### 2.2 Computed tomography scans

All patients received a contrast-enhanced CT scan in the supine position with a Big-Bore CT (Philips, Netherlands). Images were acquired from upper bound of T11 to the lower edge of ischial tuberosity, and the slice thickness was 5 mm.

### 2.3 Lymph nodes grouping

**Figure 1:**
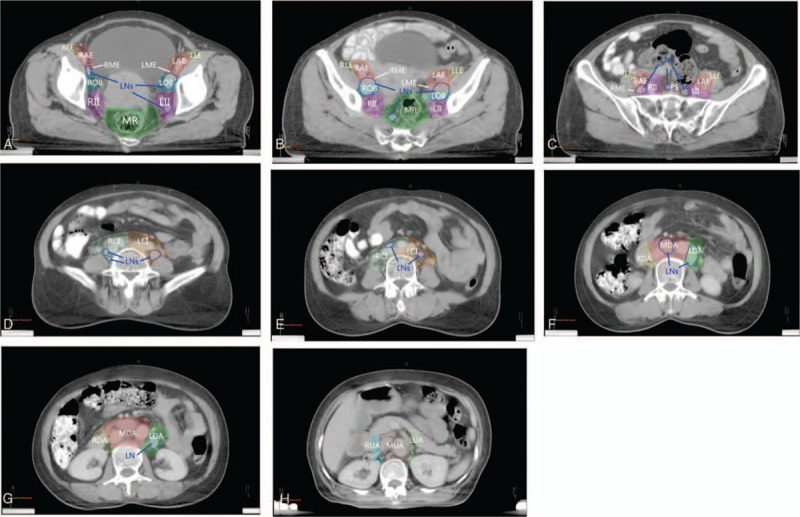
(A–H) Definition of lymph node groups. Different groups were contoured in different colors. L/R II = left/right internal iliac group, MR = mesorectum group, R/L CI = right/left common iliac group, R/M/L U/D A = right/middle/left up/down para-aortic group, PS = presacral group, R/ L M/A/L E = right/left medial/anterior/lateral external iliac group, R/L OB = right/left obturator group. Enlarged lymph nodes (LN) were contoured in blue.

Lymph nodes were assigned to a nodal group depending on their anatomic location in relation to the blood vessels. Nodal groups are defined as follows (Fig. 1):

1. Para-aortic LNs. This group was defined as all LNs adjacent to the aorta or inferior vena cava. The upper bound was top of T11 and the lower bound was aortic bifurcation. This group was divided into 2 subgroups at the level of renal vessels: up-para-aortic group (UA) and down-para-aortic group (DA). Each subgroup was further divided into right, middle, and left subgroups at right edge of inferior vena cava and left edge of aorta. So there were total of 6 subgroups: right up-para-aortic group (RUA), middle up-para-aortic group (MUA), left up-para-aortic group (LUA), right down-para-aortic group (RDA), middle down-para-aortic group (MDA) and left down-para-aortic group (LDA).
2. Common iliac nodes. This group was defined as all LNs adjacent to the common iliac vessels from the aortic bifurcation to the division of the common iliac artery into the external and internal iliac branches. This group was divided into right common iliac group (RCI) and left common iliac group (LCI).
3. Presacral nodes (PS). This group was defined as all LNs anterior to the sacrum. The upper bound was aortic bifurcation and the lower bound was lower edge of sacroiliac joints. For LNs medial to the common iliac vessels, if they were within 1 cm from the common iliac vessels, they were grouped into common iliac nodes; otherwise, they were in presacral group.
4. Internal iliac nodes. The internal iliac nodes were next to the internal iliac vessels and their branches and tributaries. This group was divided into left and right internal iliac subgroups (LII and RII).
5. External iliac nodes. The external iliac nodes surrounded the external iliac vessels until they passed through the inguinal ligament. This group was divided into left and right external iliac groups, which were further subdivided into the medial, anterior-middle, and lateral subgroups. The medial external iliac nodes were medial and directly posterior to the external iliac vein; the anterior-middle external iliac nodes were in the sulcus between the artery and vein and anteromedial to the artery; and the lateral external iliac nodes extended laterally from the external iliac artery. So there were 6 subgroups: left/right; medial/anterior/lateral; and external iliac subgroups (L/R; M/A/L; E).
6. Obturator nodes. The obturator nodes were within the triangle between the external and internal iliac vessels and were divided into right obturator group (ROB) and left obturator group (LOB). For LNs between the external and internal iliac vessels, if they were within 1 cm from the vessels, they were grouped into internal iliac or external iliac groups depending on which vessel was closer to the node; otherwise they were in obturator group.
7. Mesorectum nodes (MR). The obturator nodes were within the mesorectum region. The upper bound was the level of lower edge of sacroiliac joints. Lymph nodes above lower edge of sacroiliac joints were grouped into presacral nodes.

Lymph nodes of groups 4, 5, 6, 7 were defined as level 1 nodes, whereas groups 2 and 3 were level 2, and group 1 was level 3.

### 2.4 Measurement of LNs and definition of positive LNs

Computed tomography (CT) images were transferred and imported into Eclipse version 8.0 treatment planning software wherein all measurements were taken. The shortest axis of each lymph node was measured in a preset pelvic soft-tissue window.

CT scan was economical and practical compared with PET/CT scan, but its sensitivity and specificity were relatively low when a single cutoff value was used. In our study, two cutoff values were used. Lymph nodes with the shortest axis >1 cm were defined as positive, and those between 0.5 and 1 cm were defined as suspicious. Yang et al^[6]^ reported that the “1 cm” cutoff value corresponded to a high specificity of 96.6%. But in our study, sensitivity was as important, so a second “0.5 cm” cutoff value was used. Hilton et al^[7]^ reported that if the criterion of 0.4 cm was used, the sensitivity could be 93%. Oyen et al^[8]^ reported that, on CT images in patients with prostate cancer, a criterion of 0.6 cm resulted in a sensitivity of 78%. Using a threshold of 0.5 cm, the shortest axis diameter for metastasis (from a variety of pelvic tumors), Fukuda et al^[9]^ demonstrated a sensitivity of 85.7%. These results were comparable with PET/CT, which had sensitivity of 79% (65%–90%) reported by Havrilesky et al.^[10]^ In our study, we chose 2 cutoff values: 0.5 cm and 1.0 cm to balance between specificity and sensitivity. The results from the 2 analyses were reported separately.

Distances from the center of LNs to the edge of the nearest vessels were recorded. To minimize the bias owing to size, only LNs <1.5 cm in diameter were used to analyze the relationship between LNs and main vessels.

## 3 Results

### 3.1 Overview

In all the 244 patients, 931 LNs >0.5 cm were identified. Seven hundred ninety-five nodes were suspicious and 136 were positive in which 40 LNs had the shortest axis >1.5 cm. Sixty-three (25.8%) patients had positive LNs, 153 (62.7%) patients had only suspicious LNs, and 28 (11.5%) patients had no LNs >0.5 cm. Average number of positive and suspicious LNs per patient was 3.82 and the median number was 3.

### 3.2 Sequence of LNs metastasis

Numbers of LNs in each group were shown in Table 1. Five hundred fifty-three suspicious nodes and 104 positive nodes were in the level 1 nodes, 146 suspicious nodes and 19 positive nodes in level 2, and 96 suspicious nodes and 13 positive nodes in level 3.

**Table 1:**
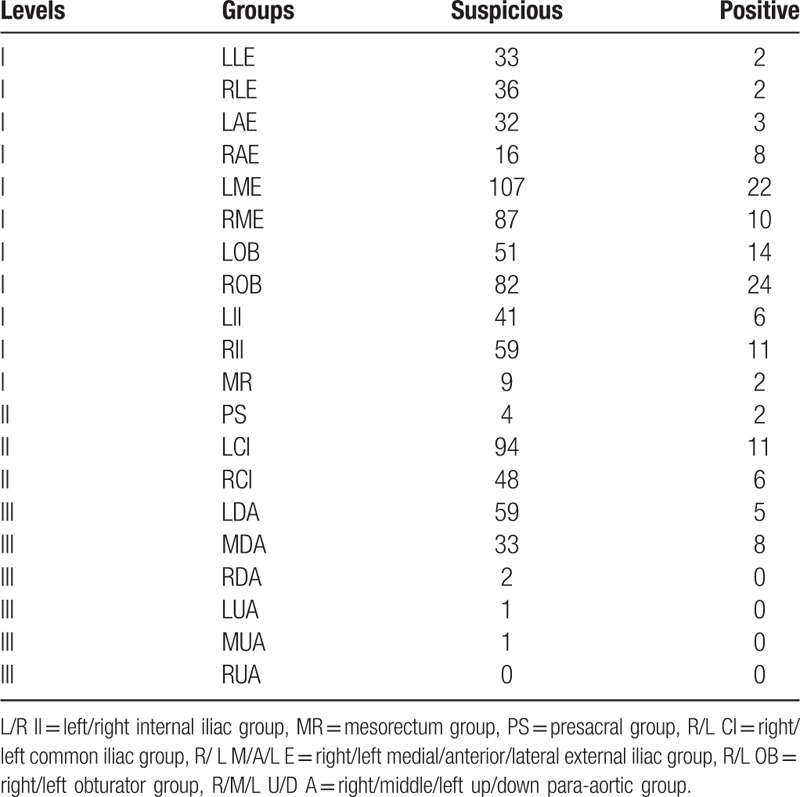
Numbers of lymph nodes in each group.

Sixty-three patients had level 1 positive metastasis and 153 patients had level 1 suspicious metastasis. Those numbers were 11 (positive) and 89 (suspicious) for level 2 and 6 (positive) and 46 (suspicious) for level 3. Relations of lymph node metastasis between levels were shown in Table 2.

**Table 2:**
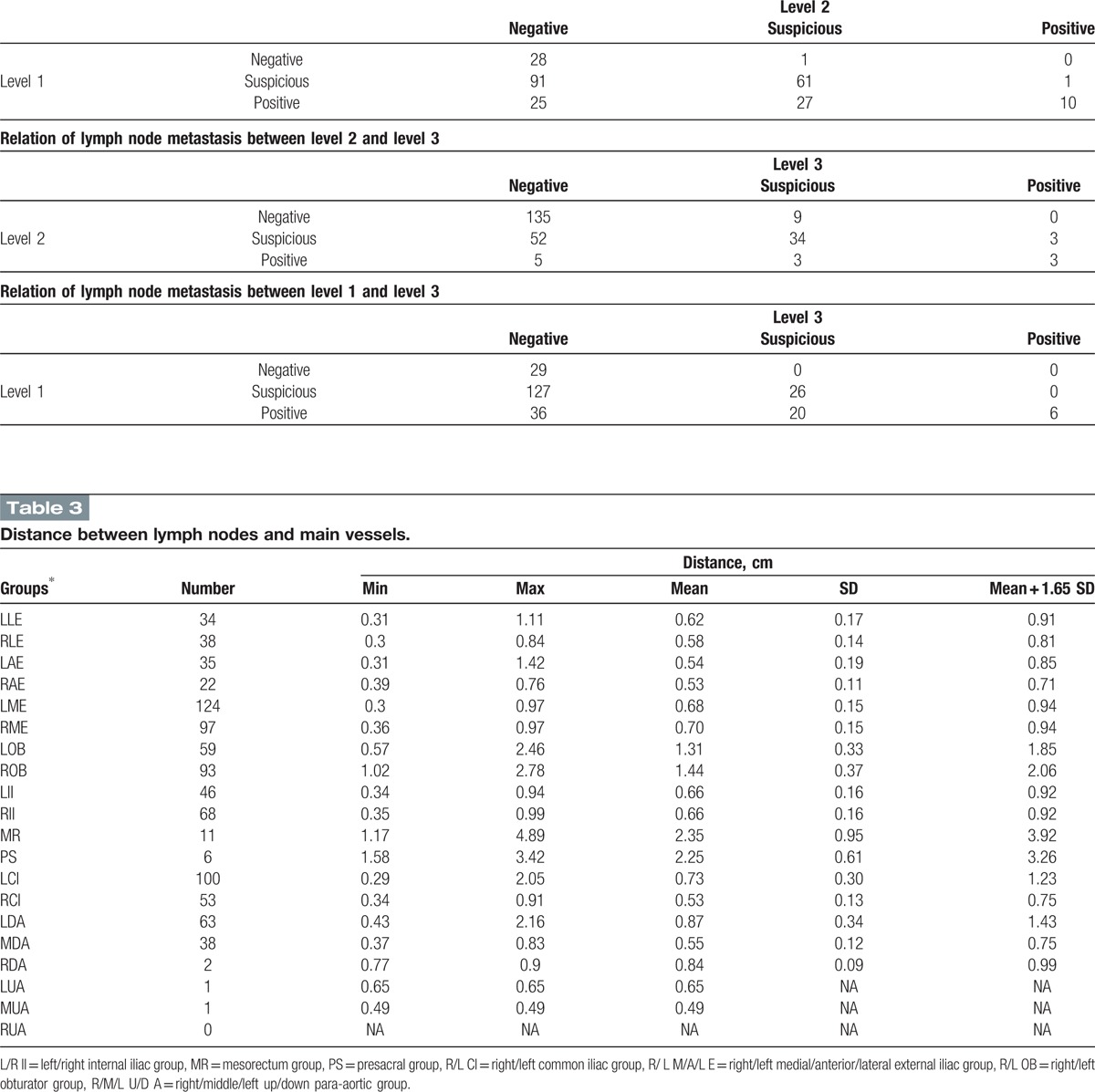
Relation of lymph node metastasis between level 1 and level 2.

Only 0.4% (1/244) of the patients had suspicious metastasis in level 2 (common iliac) without LNs found in level 1 and only another one patient had positive level 2 LNs but with only suspicious level 1 LNs. Every patient who had positive level 2 LNs had at least 1 level 1 lymph node >0.5 cm. Similar findings and relationships were noticed between level 2 and 3, and also between level 1 and 3.

### 3.3 Distance between LNs and main vessels

The distance from lymph node to the blood vessel was evaluated. In the situation where lymph node is enlarged, the distance from center of node to blood vessel might be overestimated. So 40 LNs with the shortest axis >1.5 cm were excluded from the distance analysis.

Total 891 positive or suspicious nodes were analyzed. In most of groups, LNs were within 1.0 cm of main vessels, with the exceptions of LOB, ROB, MR, PS, LCI, and LDA groups. In Iliac groups, including external and internal iliac groups, a margin of 0.9 cm CTV margin would have covered 95% of positive and suspicious nodes.

For LOB, ROB, MR, and PS groups, nodes can be relatively far away from the vessels. Adding a uniform margin from blood vessels was not an appropriate method to cover these nodes. We suggest contouring obturator CTV by joining the external and internal iliac CTV with a 1.5 to 2 cm width bar along the pelvic sidewall. A 1 to 1.5 cm width bar anterior to the sacrum might be reasonable if presacral group is part of CTV.

Table 3 shows distances between LNs and main vessels for each group. There were not enough evaluable nodes in RDA, LUA, MUA, and RUA groups to calculate proper margins from blood vessels.

**Table 3:**
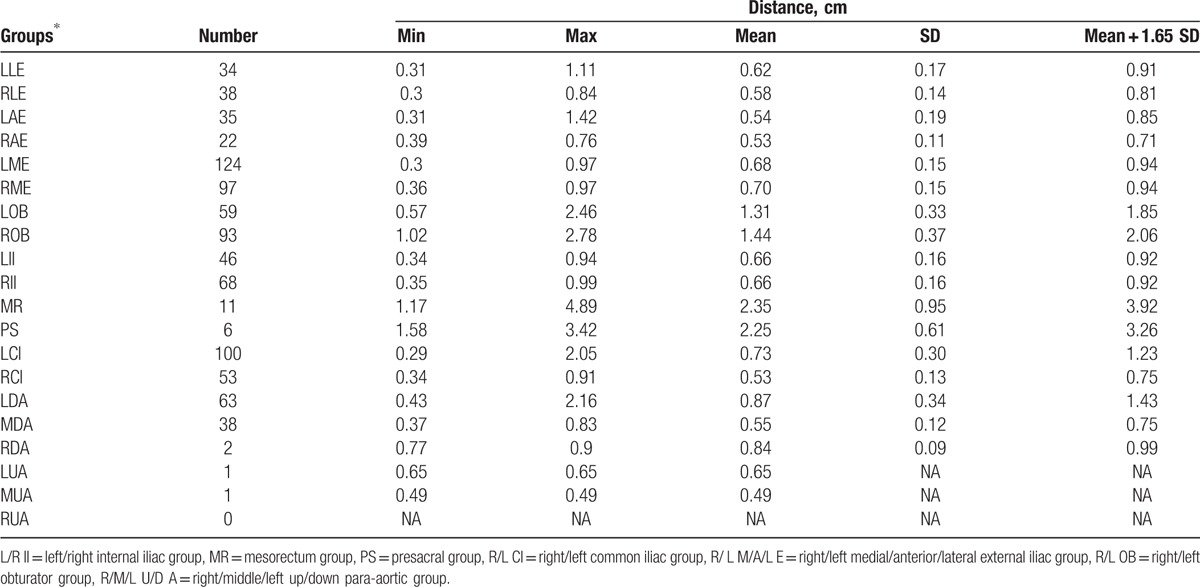
Distance between lymph nodes and main vessels.

### 3.4 Novel findings

Presacral LNs metastases were rare. Presacral nodes are typically included in CTV. In our study, only one patient had positive nodes and another had suspicious nodes in this region of 244 patients. They were the only two who had mesorectum nodes. These 2 patients had more positive or suspicious LNs than the rest of patients. Details of the 2 patients are shown in Table 4. Mesorectum and presacral nodes were more commonly involved in rectum cancer, but it was difficult to determine whether the 2 patients had rectal or mesorectal invasions based on CT. Höckel et al^[11]^ reported about 11% lymph node metastases occur in this region. However, studies based on radiography^[12–14]^ found metastases in this region were rare. In addition, recurrences in presacral region were also rare. The different findings might be because of different lymph node classification methods. It was difficult to measure exact distances between nodes and vessels during surgery, so surgeons might misclassify some internal or common iliac LNs into the presacral group. Additional study is warranted to evaluate whether presacral nodes should be part of CTV.

**Table 4:**
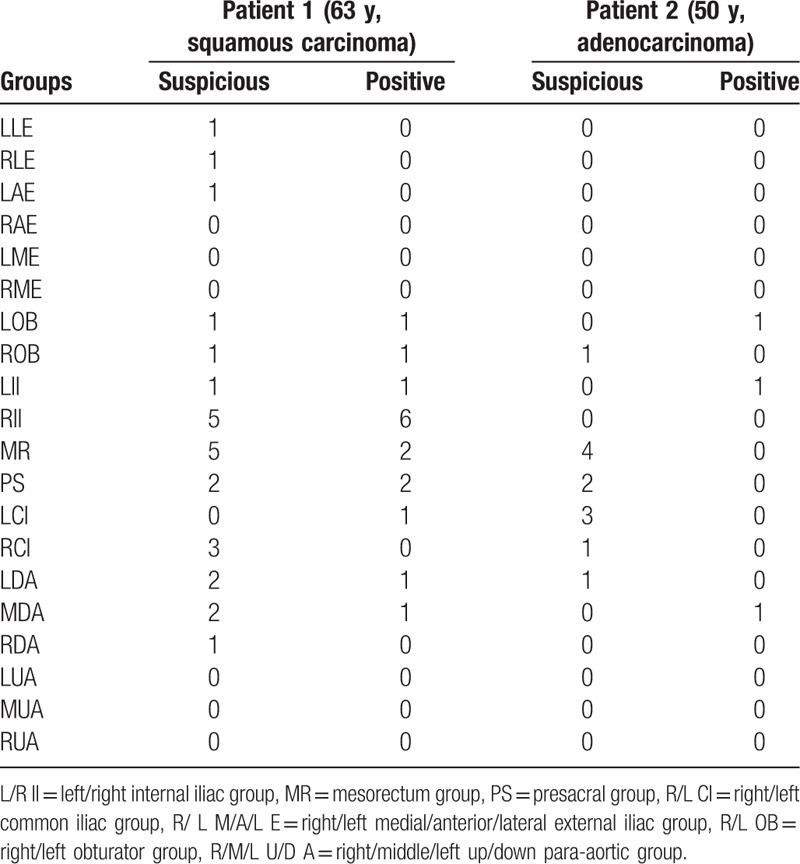
Lymph node metastasis of the 2 MR and PS-positive patients.

Left common iliac group had more nodes than the RCI. One hundred fifty-nine nodes (17 positive and 142 suspicious) were found in common iliac groups and binomial test was applied to test if nodes were distributed evenly in left and right side. Results showed the left side had more enlarged nodes than the right side (105 vs. 54, *P* = 0.00, for all nodes; 11 vs. 6, *P* = 0.33, for positive nodes; and 94 vs. 48, *P* = 0.00, for suspicious nodes). That was statistically significant and we do not have a clinical explanation for the findings. Nevertheless, it has never been reported to our knowledge.

In level 1 groups (internal and external iliac and obturator groups), 646 nodes (102 positive and 544 suspicious) were found. Left side groups seemed to have similar nodes as right side groups (311 vs. 335, *P* = 0.37, for all nodes; 47 vs. 55, *P* = 0.49, for positive nodes; and 267 vs. 280, *P* = 0.52, for suspicious nodes).

In level 3 groups, 109 nodes (13 positive and 96 suspicious) were found. Most of them located in the left and middle groups (107/109 for all nodes; 13/13 for positive nodes; and 94/96 for suspicious nodes). That means most enhanced nodes were on left of the right edge of vena cava which is consistent to other studies.^[15–17]^

Left common iliac group and left down-para-aortic group need larger margins. Different margins should be evaluated and used for different lymph node groups. In this study, we found LCI needs larger margin (1.2 cm) than the right group (0.8 cm). No previous literatures have reported this finding to our knowledge.

For para-aortic groups, a 0.8-cm margin was needed for middle subgroup, but a margin as large as 1.5 cm was needed for left subgroup, especially in the left-posterior direction. We only found 2 nodes in the right para-aortic subgroup, and whether this subgroup should be included in CTV needs further evaluation.

No additional margins were needed in anterolateral directions for external iliac groups. Taylor et al^[12]^ suggested an additional 1-cm margins anterolaterally along the iliopsoas muscle for external iliac groups, but our data did not support that argument. The maximum distances of external iliac groups were 1.11 cm for the left side and 0.84 cm for the right side in our study. In the era of conventional radiotherapy, review of lymphangiograms^[18–20]^ has shown that conventional fields miss these nodes in 34% to 45% of the cases. Despite this, the region is a rare site of recurrence. Therefore, it is reasonable to hypothesize that the distal anterolateral region (1 cm or more from the vessels) did have LNs, so they could be seen in lymphangiograms and USPIO-enhanced MRI (the technique Taylor et al used). This region mainly drains lymph of lower limbs, anal canal, and vulva regions, not of cervical or uterus regions. That is why very few enlarged nodes were found in our study and recurrences were rare even the region was not included in the treatment fields in the era of conventional radiotherapy. However, this hypothesis needs further confirmation.

## 4 Discussion

Cervical cancer is a common gynecological malignant disease, and concurrent chemoradiotherapy is the main treatment to cure locally advanced patients. Pelvic and/or para-aortic LNs metastases were common for those patients. Better understanding of lymph node metastatic pattern can help radiation oncologists to deliver precision radiotherapy, especially in the era of IMRT. However, what has been reported in the literatures^[12,13]^ was based on a limited number of patients. To our knowledge, no one has analyzed a large number of patients to provide detailed guideline on how to define CTV for locally advanced cervical cancer.

In all the 244 stage IIB patients, we found 25.8% patients had LNs metastasis, and 62.7% patients only had suspicious LNs. This is consistent to previous studies.^[21–23]^ Our study also supports the current consensus that cervical cancer metastasizes primarily to pelvic LNs through aortic lymph node metastases pathway.

There were 104 positive nodes and 553 suspicious nodes in level 1 LNs, which were far more than level 2 where only 19 positive and 146 suspicious nodes were identified. Similarly, level 2 had more positive and suspicious nodes than level 3.

Almost all patients who had enlarged LNs at a higher level group also had at least 1 enlarged lymph node at lower level; it is reasonable to state that most lymph node metastases travel from lower level to higher level. Therefore, lower para-aortic LNs should be included into CTV only when level 2 LNs are involved. If lower para-aortic LNs are involved, upper para-aortic LNs should be treated.

Obturator and medial external iliac nodes were most common nodes of metastasis in this study. We grouped these 2 regions together because a great portion of enhanced nodes was within the 2 regions. In our study, 70 positive nodes (51.5%) and 327 suspicious nodes (41.1%) were identified in these regions. This was consistent to the results of Marnitz et al^[24]^, which reported the interiliac nodes were the most common of sentinel LNs in cervical cancer.

Although a uniform margin was a relatively simple method for delineating CTV, it was no longer precise enough for modern IMRT and Image Guided Radiation Therapy. In this study, we divided LNs into groups and even subgroups and noticed different margins are needed for different subgroups. It was obvious nodes distributed unevenly inside each subgroup, and further studies are needed to improve our understanding of lymph node locations for cervical cancer.

## 5 Conclusion

Lymph node metastases are common in locally advanced cervical cancer. In our study, obturator and medial external iliac nodes were the most common area for metastases. The LCI had more enhanced nodes than the right group. Presacral nodes metastases were rare. Further study is warranted to evaluate whether this lymph node group could be excluded from nodal CTV for cervical cancer patients. The lymph node metastasizes from lower level to higher level. Different margins from blood vessels should be used for different lymph groups to define CTV.
